# Programmed death ligand-1 and CD8 tumor-infiltrating lymphocytes (TILs) as prognostic predictors in ovarian high-grade serous carcinoma (HGSC)

**DOI:** 10.1186/s43046-021-00073-5

**Published:** 2021-07-09

**Authors:** Mayada Saad Farrag, Khaled Abdelwahab, Nesrine Saad Farrag, Waleed Elsayed Elrefaie, Ziad Emarah

**Affiliations:** 1 Department of Pathology, Faculty of Medicine, Port Said University, Port Said, Egypt; 2 Department of Surgical Oncology, Mansoura Oncology Center, Mansoura University, Mansoura, Egypt; 3 Department of Community Medicine and Public Health, Faculty of Medicine, Mansoura University, Mansoura, Egypt; 4 Department of Obstetrics and Gynecology, Port Said Faculty of Medicine, Port Said University, Port Said, Egypt; 5 Department of Medical Oncology, Mansoura Oncology Center, Mansoura University, Mansoura, Egypt

**Keywords:** CD8, HGSC, Immunotherapy, PD-L1, TILs

## Abstract

**Background:**

P D-L1 is expressed in tumor cells and plays a crucial role in tumor immune escape. Tumor-infiltrating lymphocytes (TILs) as CD8 T cells contribute to reduced tumor growth. Few studies investigated the prognostic effect of PD-L1 and CD8 TILs in ovarian high-grade serous carcinoma (HGSC). In the present study, we analyzed the expression of PD-L1 and CD8 TILs in HGSC by immunohistochemistry, and results were correlated to prognosis. It was carried on 54 cases of ovarian HGSC who attended the Oncology Centre, Mansoura University, Egypt, from 2012 till 2019.

**Results:**

Nearly 60% of cases showed positive PD-L1 expression in tumor cells. Regarding the clinicopathological characteristics, higher PD-L1 expression was found among patients with residual tumor (82.4%) compared to patients with no residual tumor (54.5%), with marginal statistical significance (p 0.07). PD-L1 was significantly associated with CD8 TILs expression. Higher PD-L1 expression was found among tumors with low expression of CD8 TILs with statistically significant difference (p≤0.001). Disease-free survival (DFS) was significantly lower among the group with positive expression of PD-L1 compared to the group with negative expression of PD-L1 (p 0.01), while overall survival (OS) was not associated with PD-L1 expression. On the other hand, the overall survival (OS) in patients with high CD8 expression was significantly higher than patients with low CD8 expression (p 0.043), while DFS was not significantly different among both CD8 TILS groups.

**Conclusions:**

PD-L1 and CD8 TILs may become a promising therapeutic target for patients with ovarian HGSC. More studies are needed to further validate their prognostic effect. Precise identification of patients who will benefit from PD-L1 checkpoint blockade and TILs adaptive immunotherapy is mandatory.

## Background

Ovarian cancer is the seventh most common malignant tumor and the fifth reason for cancer deaths in females. The most common ovarian cancer type is surface epithelial tumors. Unfortunately, it is commonly diagnosed at progressive phases. Thus, it is referred to as a silent killer [1]. Serous carcinoma is the most common and the most aggressive type of epithelial ovarian cancer [2]. Serous carcinomas are separated into two distinctive types, low-grade serous carcinoma (LGSC) and high-grade serous carcinoma (HGSC) [3]. Recognition of novel molecular biomarkers may help in substantial modification of treatment improving patient prognosis [4]. Ovarian cancer frequently progresses by disseminating to the peritoneum, but how the tumor cells avoid host immunity throughout this process is not well understood [5].

Tumors escape immune recognition and actively suppress T-cell-mediated normal antitumor activity to encourage additional tumor growth and metastasis via modulation of immune checkpoints including programmed death-1/programmed death ligand-1 (PD-1/PD-L1). PD-L1 is expressed in tumor cells and has an important role in tumor immune escape and the development of a permissive immune microenvironment [6]. Some researchers recommended that PD-L1 is highly expressed in solid tumors, such as colorectal cancer, lung cancer, hepatoma, gastric carcinoma, endometrial carcinoma, and cervical cancer [6]. High expression of PD-L1 was accompanied with substantially worse overall survival (OS) in many cancers. Other researches recorded that high PD-L1 expression in tumor cells might predict promising outcomes as Merkel cell, breast, and cervical carcinomas [7, 8]. In addition, conflicting records are present regarding its importance within the same tumor type such as melanoma and non-small cell lung cancer (NSCLC) [9]. So, the prognostic values of PD-L1 for many types of cancer remain debated.

T cells react to tumor antigens with direct contribution to decreased tumor growth [10]. Essentially, the recognition of antitumor immune response in the form of intraepithelial (known as intratumoral) tumor-infiltrating lymphocytes (TILs) predicts significantly longer survival in ovarian cancer [11]. TILs can be evaluated through histopathologic and immunohistochemical examinations. CD8 is a valuable marker for TILs [12].

Few studies investigated the prognostic effect of PD-L1 in ovarian cancer particularly HGSC, and the results have been inconsistent. This may be because of different methods for assay and different cutoff values [13–15]. Thus, in the current study, we evaluated the expression of PD-L1 and CD8 TILs in HGSC by immunohistochemistry, and results were correlated to prognosis. It is possible that the biomarkers under study could guide the immunomodulatory therapy of ovarian HGSC.

## Methods

We carried out this cross-sectional study on 54 samples of primary ovarian high-grade serous carcinoma patients who attended our Oncology Centre in the period from 2012 till 2019. The cases were chosen randomly. We analyzed all patients’ clinicopathological data including tumor stage (T), lymph node (L.N) metastases (N), distant metastasis (M), staging, ascites, recurrence, residual tumor, carcinoembryonic antigen (CEA) level, cancer antigen 125 (CA125) level, and peritoneal deposits. All patients received the same combination chemotherapy protocol taxol-carboplatin either as neoadjuvant therapy (NAT) or as adjuvant therapy (AT).

Entire cases were staged by utilizing the International Federation of Gynecology and Obstetrics (FIGO) 1988 staging system and were classified also into initial stage (I–II) and progressive stage (III–IV) for the aim of statistical analysis. Also, we followed the clinical outcomes in the form of disease-free survival (DFS) and overall survival (OS). The mean duration of follow-up was 43.4 months. We calculated OS from the time of diagnosis to death. DFS is the time from the end of therapy to first recurrence or metastases.

### Immunohistochemical staining

Sections were cut from paraffin-embedded tissue blocks at 4 μm and deparaffinized with xylene, then rehydrated with graded alcohols. The primary antibodies included antibodies for PD-L1 (rabbit polyclonal anti- PD-L1 antibody, Clone GB11339, Servicebio, China) diluted 1:500, Ki-67 (rabbit polyclonal Ab, clone MIB-1, Neo Markers, USA), and CD8 (mouse monoclonal antibody, DakoCytomation, Glostrup, Denmark). We used the antibodies according to manufacturer instructions. We prepared adequate positive and negative controls concurrently with test slides. We performed antigen retrieval with heat in target retrieval solution pH 6.0 for all antibodies.

### Immunohistochemical analysis

For PD-L1, slides were assessed and scored as positive or negative for PD-L1 by utilizing a threshold of ≥1% for positive staining in tumor cells [16]. Only the membrane staining of PD-L1 in tumor cells was taken into consideration [9]. Unlike PD-1 which necessitates separate tumor, stromal, and combined scores, assessment of PD-L1 was performed by evaluating regions of staining for PD-L1 in either stromal or tumor epithelial compartment, and the average of stained cells was taken into account [17].

For Ki-67, ≥25% of positive cells was considered high expression, and < 25% was considered low expression [18]. The cutoff between the two groups was defined by the mean value of Ki-67 expression in tumor samples.

As regard CD8, each slide was screened for a hotspot of CD8+ TILs at ×20 power, within each hotspot; one high power field (HPF) at ×400 magnification was evaluated to ensure valid equally comparable areas. Only CD8+ TILs within the epithelial component of the tumor (tumor islets) were evaluated [19]. Internal positive control for CD8 was provided by stromal T cells when present. Cases were considered to have a high expression of CD8+ TILs if they had ≥10% positive staining cells/HPF while low expression of CD8+ TILs if they had < 10% positive staining cells/HPF [10]. Negative controls for all antibodies composed of patients for which the primary antibody was omitted.

### Statistical analysis

Data was analyzed by utilizing the SPSS software V.16. Categorical data was evaluated as frequency and percentage. Continuous data was evaluated as mean standard deviation (SD) or median (min-max) according to the results of Shapiro-Wilk testing for the assumptions of normal distributions of data. For statistical significance testing of continuous data, we used Welch’s t-test, or Mann-Whitney U, while chi square test or Fisher’s exact test were performed for categorical data, wherever appropriate. Kaplan-Meier test was used to test for OS and DFS of cases with regard to tumor expression of PD-L1. Comparisons were done using log rank test (Mantel-Cox). Significance level was set at 0.05.

## Results

### Clinicopathological features of all cases

The study enrolled 54 ovarian cancer cases with median duration of follow-up 40 months (min-max 3.2–87.9). Their mean age is 50.7 years (SD 9.3). The majority (74.4%) of cases presented at T stage 3/4. At presentation, about 53% of patients had no L.N metastasis and about 86% of them had no distant metastasis. About 80% of patients were FIGO stage 3/4. During follow-up, 80% had peritoneal deposits, about 61% of patients had ascites, 44% had residuals, and 40% developed metastasis. In addition, recurrence occurred in 55% of cases. Only 7 cases received neoadjuvant therapy. Death occurred in 9 patients (18%). About 60% of cases showed high expression of Ki-67.

### PD-L1 expression in correlation to other clinicopathologic features

**Fig. 1:**
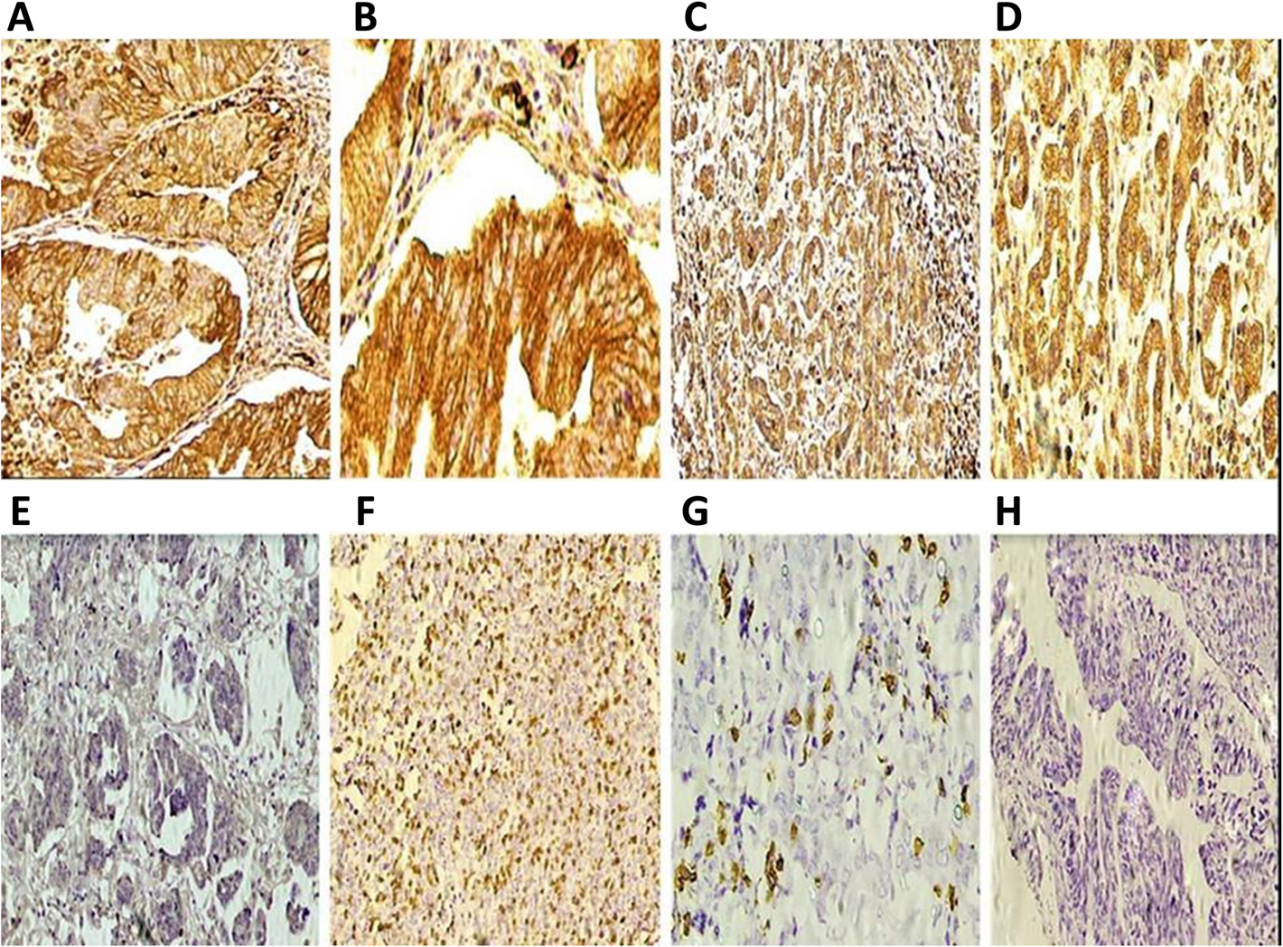
Immunohistochemical staining of PD-L1 and CD8 in different cases of ovarian high-grade serous carcinoma. **A**, **B** A case of positive PD-L1 expression showing positive membranous staining (**A** ×200 and **B** ×400). **C**, **D** Another case of positive PD-L1 expression showing positive membranous staining (**C** ×100 and **D** ×200). **E** A case of lack of expression of PD-L1expression showing negative staining ×100. **F**, **G** A case of high CD8 expression on TILs showing positive membranous staining of >10% (**F** ×100 and **G** ×200). **H** A case of no CD8 expression on TILs showing negative membranous staining ×100

Thirty-three cases (nearly 60% of cases) showed positive PD-L1 expression in tumor cells (Fig. 1A–E). The association of PD-L1 expression in tumor with different parameters of the patients is presented in Table 1. There was no statistically significant correlation among age and the PD-L1 expression (p 0.391); average age of patients who showed negative or positive expression of PD-L1 was nearly 50 years. Results showed that PD-L1 was not associated with the tumor size, L.N metastasis, distant metastasis (M), or FIGO stage on presentation. Similarly, there was no association between the development of ascites, peritoneal deposits, or recurrence, on one hand, and PD-L1 expression. PD-L1 expression was higher among patients with residual tumor (82.4%) compared to 54.5% among patients with no residual tumor, with marginal statistical significance (p 0.07). Expression of PD-L1 was not accompanied with receiving NAT (p= 1). PD-L1 expression was higher among patients with high Ki-76 but without statistical significance. Also, there was no any association with the clinical markers CEA (p=1) or CA 125 (p=0.36).

**Table 1:** The association of clinicopathological characteristics with results of PDL1 (N=54)

Clinicopathological characteristics		Negative N (%) 21 (38.9)	Positive N (%) 33 (61.1)	Significance
Age years	Mean (SD)	52.2 (7.8)	49.8 (10)	t=0.87, p=0.391^a^
Tumor stage (N=39)	T1/2	3 (30)	7 (70)	p= 1^b^
	T3/4	9 (31)	20 (69)	
L.N stage (N=28)	No	6 (40)	9 (60)	p=0.435^b^
	Yes	3 (23.1)	10 (76.9)	
M stage (N=36)	No	10 (32.3)	21 (67.7)	p= 1^b^
	Yes	2 (40)	3 (60)	
FIGO stage(N=38)	1/2	2 (25)	6 (75)	p= 1^b^
	3/4	10 (33.3)	20 (66.7)	
Ascites (N=43)	No	4 (23.5)	13 (76.5)	X^2^=1.04, p= 0.307
	Yes	10 (38.5)	16 (61.5)	
Residual tumor (N=39)	No	10 (45.5)	12 (54.5)	**X**^**2**^**= 3.33, p=0.068**
	Yes	3 (17.6)	14 (82.4)	
Metastasis (N=39)	No	6 (35.3)	11 (64.7)	X^2^= 0.29, p=0.59
	Yes	6 (27.3)	16 (72.7)	
Recurrence (N=38)	No	7 (41.2)	10 (58.8)	X^2^= 0.66, p=0.415
	Yes	6 (28.6)	15 (71.4)	
Peritoneal deposits (N= 45)	No	2 (22.2)	7 (77.8)	p= 0.695^b^
	Yes	13 (36.1)	23 (63.9)	
Neoadjuvant therapy (N=35)	No	10 (35.7)	18 (64.3)	p=1^b^
	Yes	2 (28.6)	5 (71.4)	
Ki-67 (N=53)	Low	11 (52.4)	10 (47.6)	X^2^=2.367, p=0.124
	High	10 (31.2)	22 (68.8)	
CEA (N=15)	Median (min-max)	2.8 (0.6–8)	2.1 (0.1–64)	Z=0, p=1^c^
CA 125 (N=28)	Median (min-max)	273 (170–1230)	298 (7–1268)	Z= −0.92.533, p= 0.360^c^

^a^Welch’s t-test

^b^Fisher’s exact test

^c^Mann-Whitney U

### CD8 expression in correlation to other clinicopathologic features

The association of CD8 tumor expression with different parameters of the patients is presented in Table 2. Low CD8 expression was found in 34 cases (63%), while high expression was found in 20 cases (37%) (Fig. 1F–H). There was no statistically significant correlation among age and CD8 expression (p 0.591); average age of patients who showed low or high expression of CD8 was 50.1 and 51.6 years, respectively. Results showed that CD8 was not associated with the tumor stage, L.N metastasis, distant metastasis (M), or FIGO stage. Similarly, there were no associations between occurrence of ascites, peritoneal deposits, residual, or recurrence, on one hand, and CD8 expression (p= 1, .7, .3, .8 respectively). CD8 was negatively associated with PD-L1 expression with high statistical significance (p≤0.001). CD8 expression was not accompanied with Ki-76 expression (p=0.592), CEA (p=1), or CA 125 (p=0.36). Expression of CD8 was not also associated with receiving NAT (p= 1).

**Table 2:** The association of clinicopathological characteristics with results of CD8 (N=54)

Clinicopathological characteristics		<10% N (%) 34 (63)	>10% N (%) 20 (37)	Significance
Age years	Mean (SD)	50.1 (10.1)	51.6 (7.9)	t=−0.541, p=0.591 ^a^
Tumor stage (N=39)	T1/2	5 (50)	5 (50)	p= 0.253
	T3/4	21 (72.4)	8 (27.6)	
L.N stage (N=28)	No	11 (73.3)	4 (26.7)	p=1 ^b^
	Yes	9 (69.2)	4 (30.8)	
M stage (N=36)	No	21 (67.7)	10 (32.3)	p= 1^b^
	Yes	3 (60)	2 (40)	
FIGO stage(N=38)	1/2	5 (62.5)	3 (37.5)	p= 1^b^
	3/4	20 (66.7)	10 (33.3)	
Ascites (N=43)	No	11 (64.7)	6 (35.3)	p= 1
	Yes	16 (61.5)	10 (38.5)	
Residual tumor (N=39)	No	12 (54.5)	10 (45.5)	X^2^=1.04, p=0.343
	Yes	12 (70.6)	5 (29.4)	
Metastasis (N=39)	No	13 (76.5)	4 (23.5)	X^2^=1.3, p=0.318
	Yes	13 (59.1)	9 (40.9)	
Recurrence (N=38)	No	11 (64.7)	6 (35.3)	X^2^= 0.01, p=0.899
	Yes	14 (66.7)	7 (33.3)	
Peritoneal deposits (N= 45)	No	5 (55.6)	4 (44.4)	p= 0.711^b^
	Yes	23 (63.9)	13 (36.1)	
Neoadjuvant therapy (N=35)	No	18 (81.8)	10 (76.9)	p=1^b^
	Yes	4 (18.2)	3 (23.1)	
Ki-67 (N=53)	Low	14 (66.7)	7 (33.3)	X^2^=0.287, p=0.592
	High	19 (59.4)	13 (40.6)	
PDL1	Negative	7 (33.3)	14 (66.7)	**X**^**2**^**=12.9, p≤0.001**
	Positive	27 (81.8)	6 (18.2)	
CEA (N=15)	Median (min-max)	2.1 (0.1–64)	8 (2–48)	Z=−1.22, p=0.219^c^
CA 125 (N=28)	Median (min-max)	212 (104–1268)	238 (169–1233)	Z= −0.712, p= 0.476^c^

^a^Welch’s t-test

^b^Fisher’s exact test

^c^Mann-Whitney U

### Survival results

A univariate analysis was conducted to assess the impact of PD-L1 and CD8 expression on patient’s survival. Kaplan-Meier survival curves were created, then the log-rank test.

#### Correlation of PD-L1 expression with survival

**Fig. 2:**
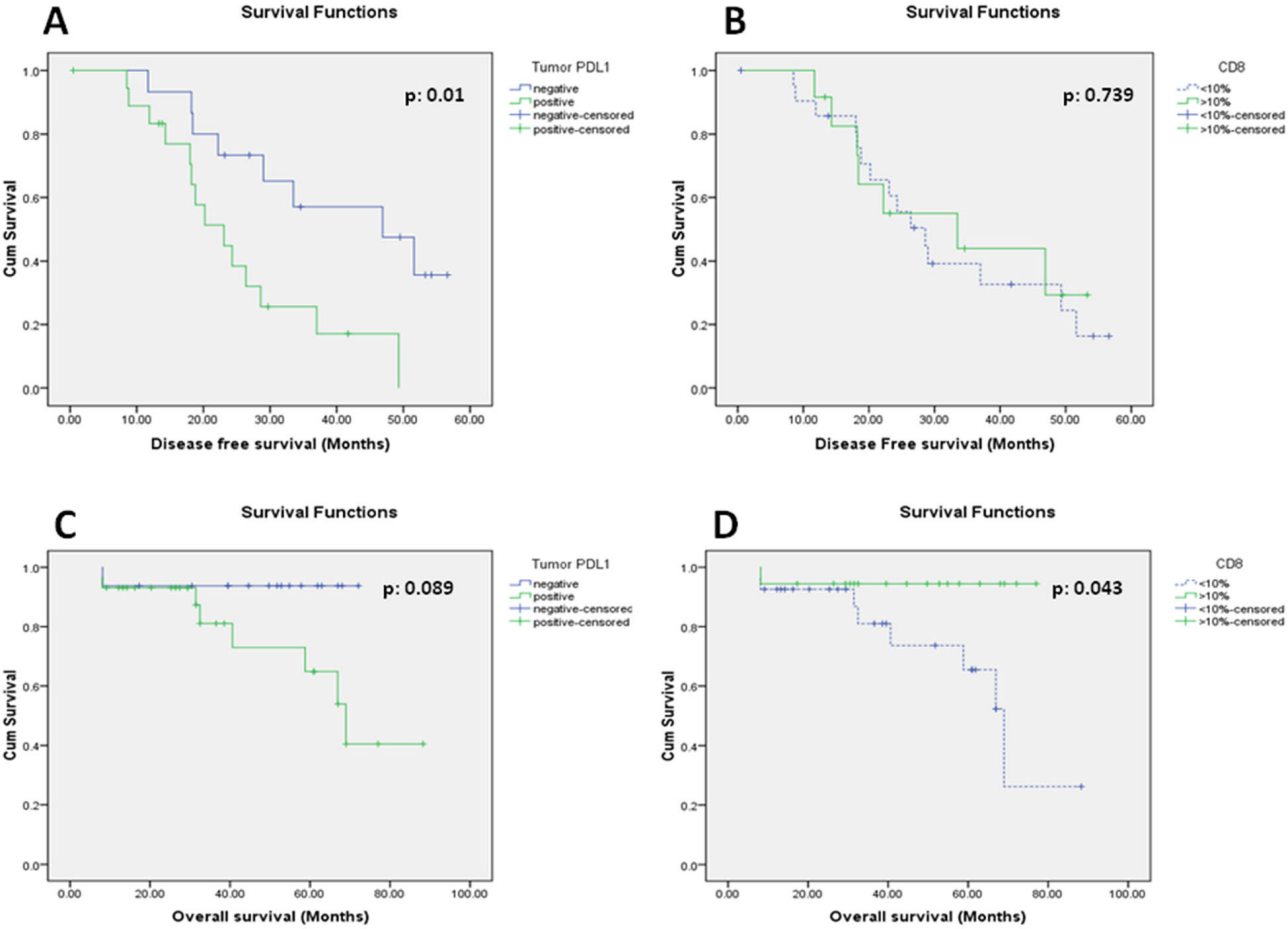
Analysis of survival in relation to tumor expression of PD-L1 and CD8 T cells. **A** Disease-free survival (DFS) and PD-L1: DFS is lower among the group with positive expression of PD-L1 (p 0.01). **B** Disease-free survival (DFS) and CD8: DFS is lower among the group with low expression of CD8<10% (p 0.739). **C** Overall survival (OS) and PD-L1: positive expression was lower than patients with negative PD-L1 (p 0.089). **D** Overall survival (OS) and CD8: expression >10% was significantly higher than patients with low CD8 (p 0.043) (Kaplan-Meier test)

Disease-free and overall survivals of the patients in relation to tumor expression of PD-L1 marker are presented in Tables 3 and 4 and Fig. 2 A and C. They show that DFS of patients was significantly lower among the group with positive expression of PD-L1 (mean, 95% CI 25.5, 18.9–32.2) compared to the group with negative expression of PD-L1 (mean, 95% CI 40.6, 31.9–49.1, p 0.011). Mean OS time in patients with positive PD-L1 expression (mean, 95% CI 64.8, 52.6–76.9) were less than patients with negative expression of the markers (mean, 95% CI 68.1, 60.5–75.7, p 0.089). However, this difference was not statistically significant.

**Table 3:** Analysis of disease-free survival (DFS) of the patients in relation to tumor expression of PDL1 and CD8 (Kaplan-Meier test)

		Total N	N of events	Censored N (%)	Survival time Mean (95%CI)	X^2a^	p
PDL1	Negative	15	8	7 (46.7)	40.6 (31.9–49.1)	6.4	**0.011**
	Positive	19	14	5 (26.3)	25.5 (18.9–32.2)		
CD8	<10%	22	15	7 (31.8)	32.04 (24.7–39.4)	0.111	0.739
	>10%	12	7	5 (41.7)	33.9 (24.1–43.7)		

^a^Log rank (Mantel-Cox)

**Table 4:** Analysis of overall (OS) of the patients in relation to tumor expression of PDL1 and CD8 (Kaplan-Meier test)

		Total N	N of events	Censored N (%)	Survival time Mean (95%CI)	X^2a^	p
PDL1	Negative	16	1	15 (93.8)	68.1 (60.5–75.7)	2.9	0.089
	Positive	29	8	21 (72.4)	64.8 (52.6–76.9)		
CD8	<10%	27	8	19 (70.4)	62.03 (49.7–74.4)	4.11	**0.043**
	>10%	18	1	17 (97.4)	73.2 (65.9–80.5)		

^a^Log rank (Mantel-Cox)

#### Correlation of CD8 expression with survival

Disease-free and overall survivals of the patients in relation to tumor expression of CD8 marker are presented in Tables 3 and 4 and Fig. 2 B and D. They show that mean OS time in patients with CD expression >10% (mean, 95% CI 73.2, 65.9–80.5) was significantly higher than patients with CD expression <10% (mean, 95% CI 62, 49.7–74.4, p 0.043). But DFS of patients was not significantly different in the group with CD expression >10% (mean, 95% CI 33.8, 24.1–43.7) from the group with CD expression <10% (mean, 95% CI 32.04, 24.7–39.4, p 0.739).

## Discussion

PD-L1 which is a co-regulatory molecule exhibits expression on the surface of different types of cells, such as immune cells as well as epithelial cells. By binding to its receptor PD-1 on lymphocytes, it generates an inhibitory signal toward the T-cell receptor (TCR)-mediated activation of lymphocytes [5]. Although PD-L1 might act as a tumor suppressor through inhibition of cancer stem cell (CSC) features in cholangiocarcinoma, tumor cell-intrinsic PD-L1 has an essential role in the promotion of cancer stemness, epithelial mesenchymal transition EMT, tumor invasion, and chemoresistance in many other tumor types. Essentially, stimulation of octamer-binding transcription factor 4 (OCT4) signaling and upregulation of EMT induce PD-L1 expression in malignant cells, thus recommending a potential immune evasion mechanism employed by CSCs throughout metastasis [6].

PD-L1 is expressed in tumor cells, and it has a critical function in tumor immune escape and in the development of a permissive immune microenvironment, via three or more mechanisms: (i) energizing tumor-reactive T cells via binding to its receptor PD-1; (ii) making tumor cells resistant to CD8+ T cell and Fas ligand-mediated lysis; and (iii) tolerizing T cells by reverse signaling via T cell-expressed CD80 [18, 19]. Moreover, PD-L1 expressed by tumor-accompanied immune cells is the main parameter responsible for tumor-accompanied immune deficiency [6].

The current study demonstrated that nearly 60% of cases showed positive PD-L1 expression in tumor cells (Fig. 1A–D). PD-L1 expression in HGSC was approached in only few studies. These studies found that PD-L1 expression was variable in cases of serous carcinoma ranging from 11 to 60% of the cases [16, 20–22].

In our study, there was no statistically significant correlation between PD-L1 expressions in tumor cells and the patient age, tumor stage, L.Ns, metastasis, or FIGO stage. These results are in agreement with a meta-analysis study conducted in 2019 and found that PD-L1 expression was not related to tumor grade, stage, L.N condition, or tumor histology [23]. In contrast to these results, Drakes et al. reported that low PD-L1-expressing cells in tumor tissue were significantly accompanied with progressive disease as well as high-grade tumors [17].

The potential causes for inconsistent outcomes may involve cancer type, tumor heterogeneity, sample size, clinical stage, several interventions, the time point of PD-L1 measurement, and the various methodologies utilized in the study (including recognition approaches and techniques) [6].

Also, we found no association between PD-L1 expressions in tumor cells and development of ascites, peritoneal deposits, or recurrence. But PD-L1 expression was higher among patients with residual tumor, 82.4% compared to 54.5% among patients with no residual tumor, with marginal statistical significance (p 0.07). In contrast, PD-L1 expression in tumor cells was accused to promote peritoneal dissemination by repressing cytotoxic T cells function in another study that found that patients with positive cytology had positive PD-L1 expression in tumor cells [5].

Expression of PD-L1 was not associated with receiving NAT (p= 1). But as known, chemotherapy induces local immune suppression in ovarian cancer via nuclear factor kappa-light-chain-enhancer of activated B cells (NF-kB)–mediated PD-L1 upregulation. Thus, an association between chemotherapy and immunotherapy targeting the PD-L1/PD-1 signaling axis might enhance the anti-tumor response and provide a talented novel therapeutic option against ovarian cancers [24]. Also, PD-L1 expression was higher among patients with high Ki-76 but without statistical significance. There was no any association with clinical markers CEA (p=1) or CA 125 (p=0.36).

Murakami et al. studied both intraepithelial and stromal CD8-positive lymphocytes and found them correlating significantly with immunoreactive type of ovarian HGSC [25]. Also, the results of Rojas and his work group found a statistically significant correlation between ovarian HGSC and CD8-positive intratumoral-infiltrating lymphocytes, but they did not establish a histopathological classification of those ovarian HGSC cases [26].

In our study, we found low CD8 expression in 34 cases of HGSC (63%), while high expression was found in 20 cases (37%) (Fig. 1F–H). Importantly, there was a negative correlation among high TILs CD8^+^ T cells and tumor PD-L1 expression with high statistical significance (p≤0.001). Such result is in accordance with a prior research which confirmed that expression of PD-L1 by tumor cells predicted paucity of intraepithelial TILs in ovarian malignant tumors [11].

There was no statistically significant correlation among CD8 expression and additional clinicopathological parameters. Such results are consistent with Adams et al.’s results who found no correlation of high intraepithelial CD8^+^ T cells with age, tumor stage, or tumor grade [10]. Also, we found that CD8 expression was not associated with Ki-76 expression, CEA, or CA 125. On the contrary, a previous result reported a significant positive correlation between expression of Ki67 and frequency of intraepithelial CD8^+^ T cells (p = .041). They hypothesized that mitotically active ovarian cancers, which are more likely to exhibit genetic instability, express a more diverse antigenic repertoire, such as neoantigens which elicit a cellular immune response [10].

High expression of PD-L1 was accompanied with considerably worse OS in cervical cancer, NSCLC, gastric carcinoma, esophageal carcinoma, glioma, and other malignant tumors. Conversely, the prognostic value of PD-L1 for particular types of malignant tumors remains debated. Particular researches recorded that high PD-L1 might predict promising outcomes [7]. Factors which affect the accurateness of PD-L1 immunohistochemistry staining are as follows. Types of antibodies utilized are different in various researches. The cutoff value of PD-L1 staining positivity is different. Also, PD-L1 expression in tumors is not uniform, and sampling time as well as site might affect the results of PD-L1 staining.

Importantly, DFS of patients was significantly lower among the group with positive expression of PD-L1 compared to the group with negative expression of PD-L1 (p 0.011). This result is consistent with a bioinformatics research which revealed that PD-L1 mRNA expression was closely accompanied with poor DFS [23]. However, that meta-analysis found that PD-L1 was not linked to DFS at the level of protein expression and suggested further studies on PD-L1 expression in tumor cells of ovarian HGSC. Thus, PD-L1 might become a talented therapeutic target for DFS of cases with this ovarian cancer. In contrast, only Webb et al. recorded that PD-L1 expression has a good prognostic effect on disease-specific survival in HGSC [16].

As regards PD-L1 and OS, we found that the mean survival time in patients with positive PD-L1 expression was less than patients with negative expression of the markers. However, this change was not statistically significant (p 0.089). Such result is in agreement with previous studies that found no correlation between the presence PD-L1 in tumor cells and OS in ovarian HGSC [17, 22, 27]. Also, a meta-analysis conducted after that suggested that PD-L1 expression was not related to OS [23].

However, few studies reported a significantly shorter OS in cases with PD-L1–positive epithelial ovarian tumors in comparison with PD-L1–negative tumors [5, 8, 10]. But in combining PD-L1 with CD8, the existence of PD-L1+ cells and CD8 TILs was associated with better prognosis than CD8 TILs alone [16]. The inconsistent outcomes of PD-L1 expression in ovarian HGSC might be owing to the various detection and scoring systems used to quantify PD-L1, differences in sample size, and temporal and spatial factors.

On the other hand, DFS of cases was not significantly different in the group with TILs CD8 expression >10% from the group with TILs CD8 expression <10% (p 0.739). But the mean OS time in cases with CD expression >10% was significantly higher than cases with CD expression <10% (p 0.043). These results are in agreement with a previous study which found that cases whose tumors had intraepithelial T cells experienced longer progression-free and OS as compared with patients whose tumors lacked intraepithelial T cells [11].

The association of antitumor immune response (intraepithelial T cells) with prolonged survival suggests that ovarian cancers are intrinsically immunogenic tumors [28]. Intraepithelial T cells and improved clinical outcome may be due to the direct function of tumor-infiltrating T cells or merely to a correlation of T cells with indolent tumors with low proliferation [10]. Optimizing approaches to select tumor-reactive TILs and expand them under optimum costimulation states which permit preferential expansion of specific T-cell phenotypes. Only a proportion of cases might be not eligible for TILs adoptive therapy, as tumors are either non-resectable or yield no tumor-reactive TILs [11].

Therapeutic potential of PD-L1 antibodies to reactivate antitumor immunity in ovarian malignant tumors is now highly considered [29]. Although clinical trials of PD-1 blockade in epithelial ovarian carcinoma are still at an initial stage, objective response rates have so far ranged from 6 to 17%. This recommends that further factors might require to be considered to more accurately predict responses [16].

The correlation of immune escape mechanisms with poor survival suggests that ovarian cancers can respond to identical immunotherapy procedures as patients with other immunogenic tumors [28]. So, antibodies which block PD-L1 or PD-1 can profoundly improve the efficacy of immune therapy.

## Conclusions

Based on the previous results, PD-L1 together with CD8 TILs may be talented therapeutic targets for cases of ovarian HGSC especially subgroups of advanced disease ovarian cancer patients. Additional studies including larger samples of patients are needed to further validate the prognostic action of both PD-L1 and TILs CD8 expression in this ovarian cancer. Thus, we can accurately identify cases that would benefit from PD-L1 checkpoint blockade and TILs adoptive therapy. Also, further studies could predict efficacy of this modality of therapy alone or in combination with other HGSC treatment modalities.

## Data Availability

All data generated or analyzed during this study are included in the article.

## Abbreviations

AT: Adjuvant therapy
CA125: Cancer antigen 125
CEA: Carcinoembryonic antigen
CSC: Cancer stem cell
DFS: Disease-free survival
EMT: Epithelial mesenchymal transition
FIGO: Federation of Gynecology and Obstetrics
HGSC: High-grade serous carcinoma
HPF: High power field
L.N: Lymph node
LGSC: Low grade-serous carcinoma
M: Metastases
NAT: Neoadjuvant therapy
NF-kB: Nuclear factor kappa-light-chain-enhancer of activated B cells
NSCLC: Non-small cell lung cancer
OCT4: Octamer-binding transcription factor 4
OS: Overall survival
PD-1: Programmed death-1
PD-L1: Programmed death ligand-1
SD: Standard deviation
T: Tumor stage
TCR: T-cell receptor
TILs: Tumor-infiltrating lymphocytes
